# Plasmon Excitations of Multi-layer Graphene on a Conducting Substrate

**DOI:** 10.1038/srep21063

**Published:** 2016-02-17

**Authors:** Godfrey Gumbs, Andrii Iurov, Jhao-Ying Wu, M. F. Lin, Paula Fekete

**Affiliations:** 1 Department of Physics and Astronomy Hunter College of the City University of New York, 695 Park Avenue, New York, NY 10065 USA; 2 Center for High Technology Materials, University of New Mexico, Albuquerque, New Mexico, 87106, USA; 3 Department of Physics, National Cheng Kung University, Tainan, Taiwan 701; 4 US Military Academy, West Point, NY, U.S.A.; 5 Donostia International Physics Center, P. Manuel de Lardizabal 4, 20018, San Sebastian, Spain.

## Abstract

We predict the existence of low-frequency nonlocal plasmons at the vacuum-surface interface of a superlattice of *N* graphene layers interacting with conducting substrate. We derive a dispersion function that incorporates the polarization function of both the graphene monolayers and the semi-infinite electron liquid at whose surface the electrons scatter specularly. We find a surface plasmon-polariton that is not damped by particle-hole excitations or the bulk modes and which separates below the continuum mini-band of bulk plasmon modes. The surface plasmon frequency of the hybrid structure always lies below 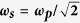, the surface plasmon frequency of the conducting substrate. The intensity of this mode depends on the distance of the graphene layers from the conductor’s surface, the energy band gap between valence and conduction bands of graphene monolayer and, most importantly, on the number of two-dimensional layers. For a sufficiently large number of layers 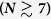 the hybrid structure has no surface plasmon. The existence of plasmons with different dispersion relations indicates that quasiparticles with different group velocity may coexist for various ranges of wavelengths determined by the number of layers in the superlattice.

Several years ago, there was considerable activity in the study of the electronic properties of layered semiconductor superlattices^1^,^2^,^3^,^4^,^5^ leading to intriguing transport and optical properties which are the result of quantum mechanical effects on the nanoscale. Specifically, it was desirable to learn how a periodic or quasi-periodic array^6^ of two-dimensional electron liquid (2DEL) layers, would lead to a modification of the response of the charge-density excitations to an electromagnetic field. Recently, there has been some investigation regarding the Coulomb excitations of an *N*-layered superlattice of free-standing monolayer graphene sheets (MGLs)^7^. However, what was neglected in that study is the interaction between epitaxial layers of graphene and a conducting substrate which may give rise to composite plasmon-plasmon resonances. A low-frequency mode emerges below the continuum of modes in the limit of a large number of layers and is associated with the air-exposed surface of the graphene-conductor substrate combination. As a matter of fact, several authors^8^,^9^,^10^,^11^,^12^,^13^ demonstrated how the electronic response properties of graphene-metal composites of Ru and Ni, for example, are much different from free-standing structures. Furthermore, these complex carbon/metal interfaces are interesting because of the unusual and fundamental physics regarding their electronic and magnetic properties at the two-dimensional (2D) interfaces. Examples of these systems occur in intercalated graphite^14^, incommensurate transition metal/graphene^15^ and carbide/graphene interfaces^16^. Possible motivation for pursuing this area of research is the tunability of graphene plasmons by a substrate which is a promising emerging field of graphene-based plasmonics.

Here, we demonstrate how the intensity of the response of the surface plasmon arising when *N* graphene layers interact with a conducting substrate may be adjusted by changing the layer-substrate separation, the energy band gap or the number of 2D layers. It has been shown that such an energy gap may be produced by circularly polarized light^17^,^18^. However, there are a number of methods for creating an energy bandgap for a graphene layer, including the use of various substrates. But, while we present and discuss these mechanisms in our paper, we emphasize circularly-polarized light irradiation because of its wide ranging tunability where the energy gap may go up to 100 *meV* being modified with intensity of the light^19^, and also because this arrangement does not lead to any additional plasmon damping in graphene which might not be the case while using certain types of substrates^20^,^21^,^22^.

We employ our model to determine the plasmon excitation dispersion relation for a single layer on a conducting substrate and show how our results may simulate the experimental data recently reported by Politano *et al*.^9^,^10^,^11^,^12^,^13^. Additionally, this paper investigates multi-layer graphene generally, showing how its new surface mode depends on the in-plane wave vector and the critical wave vector *q*_*c*_ which marks the onset of damping by bulk modes in the miniband continuum. If the distance between the layer and the surface is decreased to several nanometers, our model faithfully reproduces the finding recently reported experimentally for nonlocal plasmon branches. Consequently, this successful agreement between our theory and the experimental data for one layer motivated us to consider a superlattice of graphene layers and the effect of the plasmon continuum on each separate mode.

There is a number of important real-world modern applications of plasmons in graphene-based heterostructures which definitely should be pointed out. A comprehensive review describing these applications could be found in ref. 23. Recently, graphene was combined with prefabricated metamaterials and plasmonic nanoarray, which led to the creation of tunable *hybrid* optical tools. Consequently, it is of the highest importance to obtain full information about the behavior (primarily, dispersions and damping) of the plasmon modes interfaced with the substrates of a different material^24^,^25^. Graphene-metal contacts are crucial components for each graphene-based device. Therefore, detailed information about the behavior of such plasmon modes, primarily dispersion relations and damping, is a key step toward designing the plasmonic applications of graphene^26^,^27^. Surface plasmon polaritons in graphene metals have been used to create biosensors. Apart from that, interfacing graphene with a metal may also introduce some unexpected properties, such as superconductivity, magnetism and formation of Schottky barriers at the interface^28^,^29^.

The model we use for a superlattice consists of *N* 2D graphene layers whose planes are perpendicular to the *z*-axis at 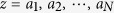 and the substrate occupies the half-space *z* < 0. Each graphene layer will be described by an energy band structure for Dirac fermions and may be intrinsic, gapped or doped. This model does not allow inter-layer hopping and hence it differs from the one we employed to describe the anisotropy of *π*-plasmon dispersion in AA-stacked graphite^30^. The screening of an externally applied frequency-dependent potential by the polarized medium requires a knowledge of the dielectric function of the structure which we obtain in the random-phase-approximation (RPA). We present our method of calculation in Section 0. Section 3 is devoted to numerical results and discussion of our plasmon dispersion when several graphene layers interact with a conducting substrate. The simulated data show how the intensity of the modes depends on the number of layers which are stacked, their distance from the conducting surface as well as the energy gap. For *N* = 1 gapless MLG, when the conductor surface-MLG separation exceeds a critical distance *d*_*c*_, the intensity of the surface plasmon in the long wavelength regime is sufficiently high to be observable up to some cut-off wave vector *q*_*c*_ of the surface plasmon frequency. Beyond *q*_*c*_, the intensity of *ω*_*c*_ is very weak until the plasmon wave vector reaches some value 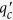. However, when the surface-MLG separation is less that *d*_*c*_, the surface plasmon intensity in the long wavelength regime is weak and the mode only appears at shorter wavelengths when the in-plane wave vector 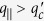. Interestingly, for gapped graphene, *ω*_*c*_ is completely suppressed when the surface-layer separation is less than *d*_*c*_. We then make some concluding remarks in Section 4 regarding the inspiration for our work, a summary and significance of our findings, and what new theoretical formalism is presented in our paper.

## General Formulation of the Problem

In our formalism, we consider a nano-scale system consisting of an arbitrary number of 2D layers and a thick conducting material. The layer may be monolayer graphene or a 2DEL such as a semiconductor inversion layer or HEMT (high electron mobility transistor). The graphene layer may have a gap, thereby extending the flexibility of the composite system that also incorporates a thick layer of dielectric material. The excitation spectra of allowable modes will be determined from a knowledge of the nonlocal dielectric function 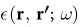 which depends on position coordinates **r**, **r**′ and frequency *ω* or its inverse *K*(**r**, **r**′; *ω*) satisfying 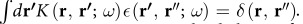.

In operator notation, the dielectric function for the *N* 2D layers and a semi-infinite structure is given by^31^


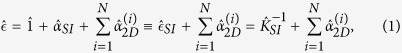


where 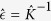 with 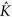 the inverse dielectric function satisfying


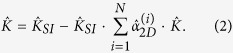


In integral form, after Fourier transforming parallel to the *xy*-plane and suppressing the in-plane wave number *q*_||_ and frequency *ω*, we obtain


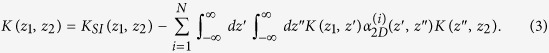


Here, the polarization function for the 2D structure is given by


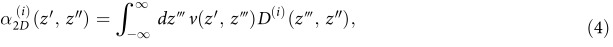


where the 2D response function obeys


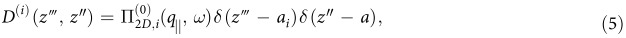


with 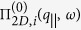 the single-particle in-plane response^32^,^33^,^34^,^35^. The 2D RPA ring diagram polarization function for graphene with a gap Δ may be expressed as


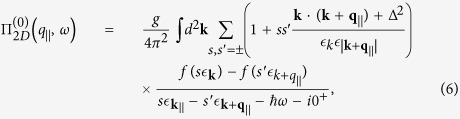


where *g* = 4 takes account of valley and spin degeneracy. At *T* = 0, the Fermi-Dirac distribution function is reduced to the Heaviside step function 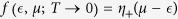. In the long wavelength limit, the real part of 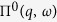 is given by


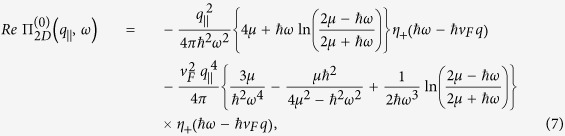


and the imaginary part by


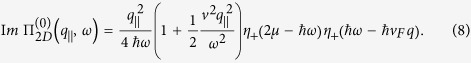


We clarify that our long wavelength approximation of 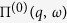 was only used to estimate the plasmon frequency within a region where the plasmon is well-defined (region 1*B* in ref. 32). From an analytic point of view, we may only consider and estimate the frequency of well-defined plasmons with negligible or no damping, i.e., *γ* ≪ *ω* in Eq. (19). If a plasmon branch enters a particle-hole region, the plasmon is overdamped, not well-defined, and its frequency cannot be estimated in this way. The Landau damping due to single-particle excitations is partially responsible for one not observing the lowest mode for small separations between the 2D layers and the substrate. This behavior was discussed in detail in our paper and seems like an explanation for the experimental data we referred to. It is also worthy noting that our analytic expressions for the polarizability in Eqs (7) and (8) include the next order ≃*q*^4^ of the corresponding results in ref. 32 provided that *ω* > *v*_*F*_*q*.

It is crucial to highlight the fact that our numerical simulations were based on the full nonlocal polarization function. It is well-known that for graphene, and especially for gapped graphene, the polarization function is determined as a piecewise function with different expressions for the regions of *q* − *ω* domain. The point is that we have written the expression for a region where the plasmon is not damped. If a plasmon branch is located below the diagonal *ω* = *v*_*F*_*q*, the plasmon is strongly damped because of the singularity 
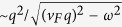
 of the intra-SPE. The frequency of such plasmons could not be obtained, even approximately, using wave number expansion techniques since the condition of a well-defined plasmon *γ* ≪ *ω* is not satisfied. We provide the long-wave limit approximation for the polarization function only in the region where the plasmon is undamped or almost undamped. We again emphasize that we employed the full expression for the one-loop electron polarization function, which is valid at arbitrary wave vector and chemical potential for both gapless and gapped graphene to obtain all our numerical results and graphs. Both intra- and inter- band transitions have been taken into account.

Our model does not include effects due to plasmon-plasmon scattering, a higher order effect. Also we employed Dirac electrons for both gapped and gapless graphene, since the doping is not high enough to be considered in the anisotropic region. These approximations are applicable for low energy plasma excitations and sufficiently clean samples. All relevant theoretical papers on plasmons in graphene and two-dimensional electron liquid, such as refs 32,33,35,36 were based on these considerations.

Upon substituting this form of the polarization function for the monolayer into the integral equation for the inverse dielectric function, we have


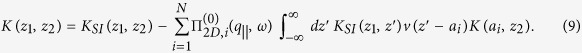


We now set *z*_1_ = *a*_*i*_ in Eq. (9) and obtain


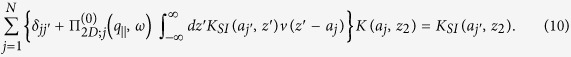


These linear algebraic equations may be solved simultaneously and their solutions expressed in matrix form as


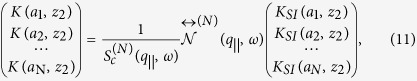


where 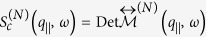 with matrix elements given by


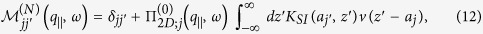


and 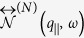 is the transpose cofactor matrix of 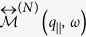.

In mean-field theory, we have^37^


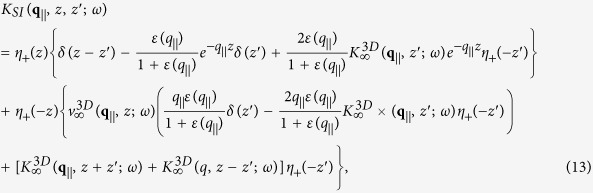


where


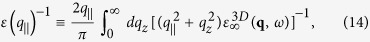


and 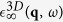 denotes the three dimensional (3D) bulk dielectric function of the thick-slab material. Furthermore, Eq. (13) introduces the definitions


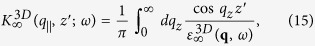


and


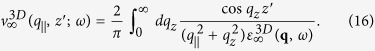


Making use of these results in Eq. (11), we then obtain


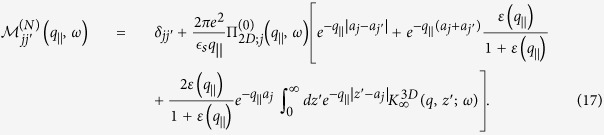


If the bulk plasma within the semi-infinite slab is fully local in the sense that 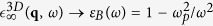, in terms of the bulk plasma frequency *ω*_*p*_, then we use


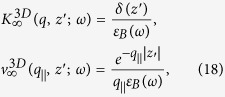


from which the corresponding local inverse dielectric function 
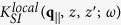
 may be obtained using Eq. (13).

While Eq. (17) is useful because of its apparent simplicity, it is necessary to understand that its validity is restricted because the *q*_*z*_-integrations in Eqs (14) through (16) extend over an infinite integration range. This blends the effects of the boundary/image length scale with that of the *q*_*z*_-nonlocality dependence, eliminating the possibility of an unrestricted limit *q*_*z*_ → 0 and modifying the plasmon dependence on *q*_||_. Additionally, the imaginary part of 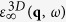 is accounted for in these *q*_*z*_-integrations, which consequently contributes to damping of the surface plasmon modes even in the low-*q*_||_ limit. The “nonlocal” *q*_||_-correction to the surface plasmon, and its imaginary part involving damping, depend on the properties of the bounding surface. But, there is a range of applicability. This can be seen by examining the parameter measuring the importance of nonlocality in the bulk dielectric function 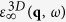, namely 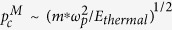, where the characteristic thermal energy *E*_*themal*_ = *μ* is the Fermi energy in the degenerate substrate with electron effective mass 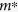. For 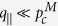, it is reasonable to neglect nonlocality, at least in the surface plasmon frequency 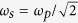 as well as in comparison with other more pertinent sources of nonlocal behavior (but it cannot be neglected in the damping of the surface plasmon).

## Numerical Results

**Figure 1:**
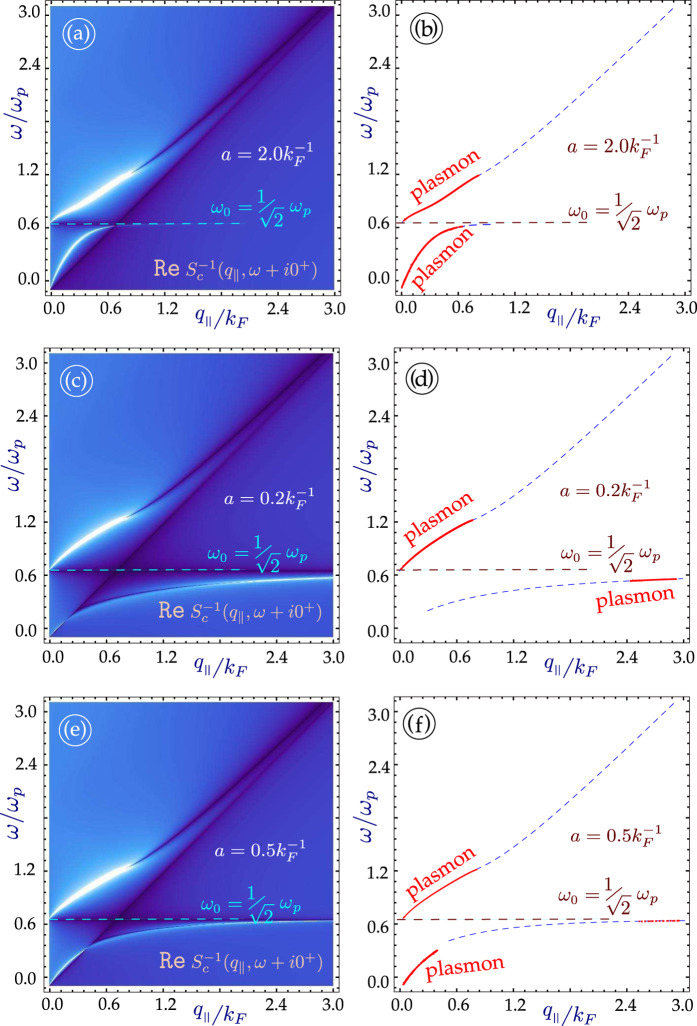
Plasmon dispersion relation for a semi-infinite conductor which is Coulomb-coupled to monolayer graphene for various surface-to-layer separations. In the panels (**a**,**c**,**e**) on the left-hand side, we present density plots of the inverse dispersion function 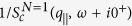 with peaks corresponding to the undamped plasmon modes. The right panels (**b**,**d**,**f**) show numerical solutions for the plasmon branches, both Landau damped and undamped. The distances chosen are *ak*_*F*_ = 0.2, 1.0 and 0.5, correspondingly. All plots are provided for extrinsic graphene (doped) with zero energy bandgap.

In this section, we present numerical results for the plasmon dispersion for a system consisting of a semi-infinite conducting medium which is Coulomb-coupled to *N* = 1, 2, 4 layers of graphene as shown in Figs 1 through 4. We note that both the plasmon solutions and damping by bulk modes in the miniband continuum crucially depend on the separation between the constituents as well as the energy gap between the valence and conduction bands. For a single layer, our results shown in Fig. 1 demonstrate that if the plasmon mode enters a region with 
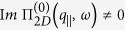
, the mode is Landau damped. Our calculations also show that when the distance *a* is less than a critical value 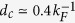, in terms of the Fermi wave vector 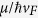, the lower acoustic plasmon mode is over-damped near the origin and this behaviour seems analogous to data reported experimentally^9^,^10^,^11^,^12^,^13^. This is obviously the case if the plasmon branch goes below the main diagonal 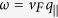. The damping, as well as the critical distance changes in the presence of an energy bandgap for graphene.

**Figure 2:**
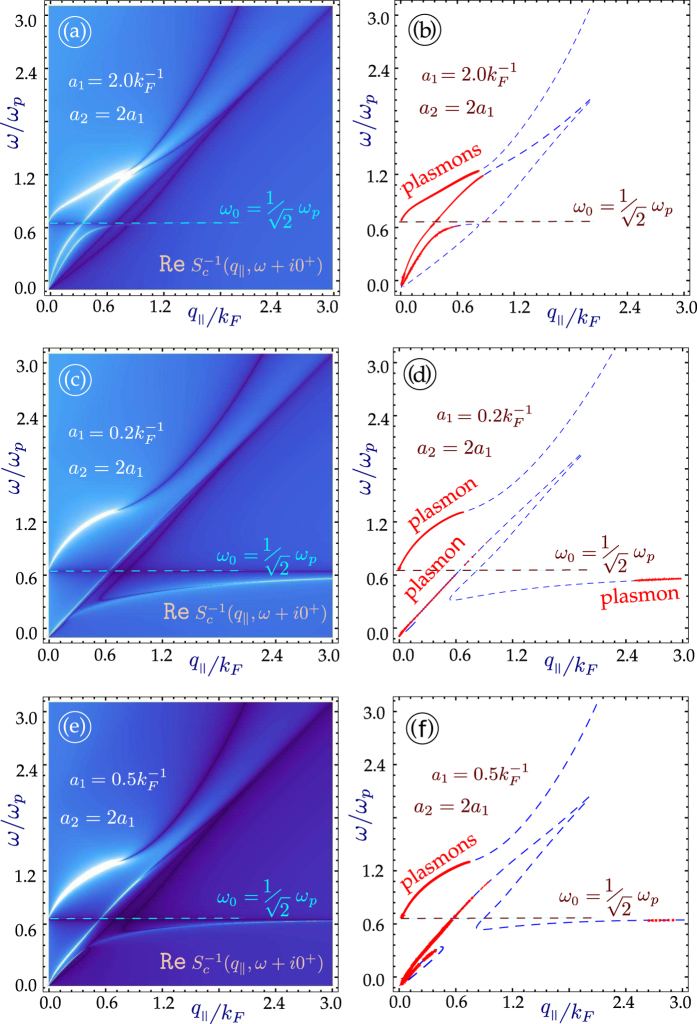
Plasmon excitation spectra for a semi-infinite conductor interacting through the Coulomb interaction with *N* = 2 monolayer graphene sheets located at chosen distances from the surface. The left-hand panels (**a**,**c**,**e**) give density plots of the inverse dispersion function 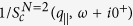 with peaks corresponding to the plasmon modes. The right panels (**b**,**d**,**f**) show the numerical solutions for the Landau damped (dashed blue lines) and undamped (red curves) plasmon branches. The plots show results for various distances between the surface and the layers: *a*_1_*k*_*F*_ = 2.0, 0.2 and 0.5, respectively. The second layer is placed at a distance *a*_2_, equal to twice as large as *a*_1_. All the plots are provided for extrinsic graphene (doped) with zero energy bandgap.

Similar conclusions for a pair of graphene layers electrostatically coupled to a semi-infinite conducting material are presented in Fig. 2. The principal difference between the case when there are two Coulomb-coupled layers is that if the distance of the layer nearest the conductor is less than the critical separation *d*_*c*_, both symmetric and antisymmetric modes become damped, for different ranges of wave vector. We emphasize that the upper plasmon branch (symmetric mode) remains almost unchanged for all cases, either with one or two graphene layers.

In order to observe the plasmon coupling we must ensure that the surface mode is not damped due to the inter-band single particle excitation, i.e., the surface plasmon frequency is comparable with the Fermi energy in graphene. The latter is in the range of the graphene 2D plasmon energy, i.e., let’s say below 1 *eV*. Indeed, very recently, Politano and collaborators have published a joint experimental-theory paper on *Bi*_2_*Se*_3_ which is a heavily doped topological insulator, where it seems that the surface plasmon energy is 104 *meV*, around 0.1 *eV*. High-resolution electron energy loss spectroscopy has provided evidence of mutual interaction between the surface plasmon and the Dirac plasmon of *Bi*_2_*Se*_3_^38^. Additionally, at the graphene/*Bi*_2_*Se*_3_ interface, which was recently experimentally realized by Kepaptsoglou *et al*.^39^, the surface plasmon of *Bi*_2_*Se*_3_ should hybridize with plasmons in graphene.

On the other hand, the zero-temperature doping Fermi energy in graphene can also vary significantly depending on the electron density and may be estimated as^36^ 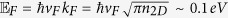 for 
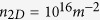
. Consequently, it is more justified to consider a conductor with the possibility to vary the bulk plasmon frequency rather than a specific metal. However we can always achieve a situation in which the surface plasmon branch is located away from the interband single particle excitation region and, therefore, is not Landau damped.

Regarding the units we used to set our length scale by, we would like to make the following remarks. Presenting the inter-layer distances in Angstroms or nanometers would immediately allow an experimentalist length scales to work with. The Fermi wave vector in graphene is determined directly from the electron density and for a standard value 
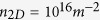
 it could be estimated as 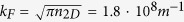. Consequently, the typical distance between the 2D layer and the surface or between neighboring 2D layers (the unit) is *a*_0_ = 6 *nm* (nanometers) and the estimated critical distance at which the lower plasmon branch no longer exists in the long-wavelength limits is *a*_*cr*_ = 2 *nm* (nanometers) or ~10^1^ Å. However, these distances could easily vary by an order of magnitude, or even more, by changing the electron density in graphene, which gives enough flexibility for experimental arrangements. For this reason, we chose to set our scale in terms of *k*_*F*_ in all our graphs. Comparison with analytical results for a single layer of graphene on a metal surface is helpful. The spectra of plasma waves in gated graphene heterostructures were addressed for various voltages and temperatures^40^. As a matter of fact, an interesting outcome is that the plasma wave velocity might exceed that of standard semiconductor-based heterostructure. Also, a number of unusual and intriguing many-body phenomena have been revealed. These include long-lived oscillations at long wavelengths^41^. However, we must emphasize again that our numerical results for hybridized plasmons were based on a novel mean field theory formalism which has never been employed or reported elsewhere. Each plasmon mode has a finite lifetime and in the case of weak dissipation, one may calculate the dissipation rate *γ*(*q*, *T*; *μ*) (inverse lifetime) with the use of


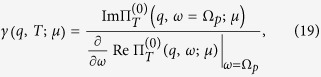


where the evaluation of the plasmon dispersion *ω* = *Ω*_*p*_(*q*) is a prerequisite for calculating *γ*(*q*, *T*; *μ*). This is basically obtained by having 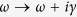 in the Drude model. This leads to broadening of the plasmon spectrum^42^,^43^,^44^,^45^,^46^,^47^.

As far as the presentation of the plasmon peaks is concerned, it is important to note that each density plot with a peak corresponding to a plasma mode is accompanied by a line plot in which an exact numerical solution of the dispersion equation is obtained for each branch, either damped (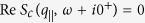) or undamped 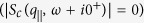, which contains all the crucial information about the nonlocal plasmon modes.

One of the principal approximations in our numerical calculations is that we treated the surface plasmon mode in the local limit where it is free from damping, whereas the acoustic plasmon attributed to the graphene layer was presented within a nonlocal formalism which includes damping. The approximation is reasonable because the surface plasmon frequency is well separated from the bulk single-particle excitations and is undamped over a wide range of wave vectors, thereby making the bulk dielectric function 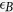 a justifiable approximation for 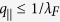 where the Fermi wavelength in metals is comparable to the lattice constant 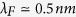.

**Figure 3:**
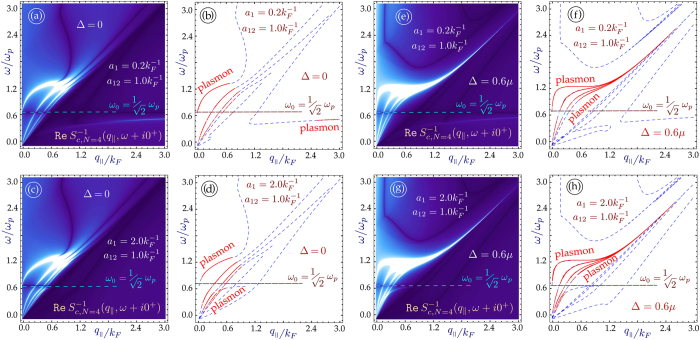
Density plots showing the bulk modes in the miniband continuum and the line traces of plasmon dispersion for *N* = 4 graphene layers on a conducting substrate. The line plots show damped (dashed blue lines) and undamped (red curves) plasmon excitations. In (**a**–**d**), the layers are gapless, and in (**e**–**h**) each layer has a gap Δ = 0.6 *μ*. The layers are equally spaced with inter-layer spacing 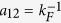. The separation between the first layer and the surface was chosen as *a*_1_*k*_*F*_ = 2.0 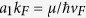. If the gap or number of layers is increased, the lowest branch does not re-appear for large *q*_||_.

**Figure 4:**
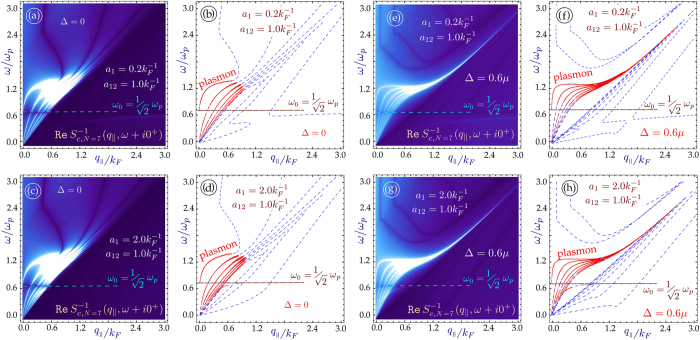
Plasmon dispersion for *N* = 7 graphene layers on a conducting substrate. As in Fig. 3, the layers in (**a**–**d**) are gapless, whereas in (**e**–**h**) each layer has a gap Δ = 0.6*μ*. The inter-layer separation is 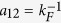. The first layer is at distances *a*_1_*k*_*F*_ = 0.2 from the surface of the semi-infinite conductor. If the gap or number of layers is increased, the lowest branch does not re-appear for large *q*_||_.

The role played by the energy band gap is an important part of our investigation. For monolayer graphene, an energy gap leads to an extended region of undamped plasmons^33^. As we mentioned before, we pay particular attention to the regions outside of the single-particle excitation continuum with 
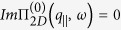
, since the plasmons in these regions are not Landau-damped. In Figs 3 and 4, we have plotted the plasmon dispersion relation for *N* = 4 and *N* = 7 graphene layers without and with an energy bandgap as well as for various distances between the nearest layer to the conducting surface. These results show that for a conducting substrate surface plasmon frequency denoted by 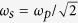, the surface plasmon frequency *ω*_*c*_ of the hybrid superlattice always lies below *ω*_*s*_. Furthermore, the intensity of this mode depends on the distance of the graphene layers from the surface of the conductor as well as the energy band gap between the valence and conduction bands of MLG. In the absence of a gap, our calculation shows that when the conductor surface-MLG separation exceeds a critical distance *d*_*c*_, the intensity of the surface plasmon in the long wavelength regime is high and may be detected up to some cut-off wave vector *q*_*c*_. For *q*_||_ > *q*_*c*_, the intensity of *ω*_*c*_ is very weak until the plasmon wave vector exceeds some value 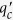. However, when the surface-MLG separation is less than *d*_*c*_, the surface plasmon intensity in the long wavelength regime is weak and the mode only appears at shorter wavelength when 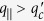. For gapped graphene, the surface plasmon frequency *ω*_*c*_ is completely suppressed when the surface-layer separation is less than *d*_*c*_.

## Concluding Remarks

The appearance of a surface plasmon polariton for a multi-layer structure consisting of 2DELs was predicted in the 1980’s by Giuliani and Quinn^2^ as well as by Jain and Allen^3^. This surface mode is free from Landau damping or damping by bulk modes in the miniband continuum and lies “*above*” the continuum of bulk modes. Additionally, this surface mode only exists above a critical wave vector 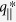 which is determined by the layer spacing and the difference in the background dielectric constants of the layers and the surrounding medium. When 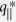, there is damping by the bulk plasmon modes. As a matter of fact, the dispersion equation for the layered superlattice structures investigated in^2^,^3^ is a special case which may be obtained from our more general Eq. (17) where we included the effects arising from a substrate.

Very recently, in a series of experiments to determine the nature and behavior of plasmon excitations in graphene interacting with metallic substrates, Politano *et al*.^9^,^10^,^11^,^12^ showed how the dispersion and intensity may be affected in a substantial way. The experimental results show that self-doped graphene supported by a metal substrate has two plasmon branches. There is an acoustic plasmon, with a linear dispersion, and a nonlinear plasmon. Both plasmon branches are similar in nature to those we presented in Fig. 1, originating from the presence of a substrate. The present paper investigating the effects of a substrate on the plasmon excitations in a superlattice of graphene was stimulated by the experimental results on supported graphene. We are aware of the theoretical work on free-standing superlattice structures of MLGs^7^, but the results there do not address the coupling to a metallic substrate which drastically affect the plasmon dispersion relation as may be observed from Figs 1 through 2.

It should be recalled that in our previous work of ref. 31, we found plasmons in a region which is devoid of collective charge density oscillations for free-standing graphene, thereby showing the influence a conducting substrate has on the charge Coulomb excitations. In our paper, it should be recognized and emphasized that we have found regions where now there is no Landau damping of the coupled plasmons, and this crucially depends on the number of Coulomb-coupled graphene layers with each other and with the substrate. This unexpected discovery may only be achieved through careful and tedious numerical simulation experiments, becoming even more challenging as the number of two-dimensional layers is increased. Indeed, other authors^7^ obtained predictably many branches for a superlattice of graphene layers and so did the classic papers of Giuliani & Quinn^2^ as well as Jain and Allen^3^.

For clarity, we wish to highlight our main results. We have found that the lowest interface layer plasmon branch is separated from all the others and exists in very distinctive regions which are free from Landau damping. Its behavior is determined not only by the damping and the distance from the substrate, but also by the continuum of the other plasmon modes. This result was neither encountered for one graphene layer not Coulomb-coupled to a conductor (ref. 37) nor for a superlattice of free-standing graphene/semiconducting 2D layers. Additionally, our calculations have shown that an energy bandgap is an influential factor affecting both the plasmon mode dispersions and its damping. All cases which we investigated are unique and provided new information about the nonlocality of plasmon excitations for the structure. An upshot of our novel theoretical formalism for an arbitrary number of 2D layers Coulomb-coupled to a conducting substrate is the closed-form analytical result we derived for the plasmon dispersion equation which may now be used in studies involving other types of 2D materials such as silicene and germanene. The way in which a substrate affects correlation energy is another possible application for our formalism. We have confidence in our model calculations since the compared numerical results for one layer of graphene on a metal substrate showed remarkable similarity with the experimental data for monolayer graphene on a substrate such as platinum or rubidium^23^. Consequently, our predicted results for the behaviors of Coulomb excitations for a multi-layer structure would be great help for experimentalists seeking to interpret their data or even design an experiment.

As we emphasized above, we have carried out extensive numerical simulations for the dispersion relation of Coulomb excitations in hybrid structures consisting of many graphene layers and we are making predictions about their dispersive properties. As far as we are aware, there are no reported experimental data which we can use for comparison. However, as we indicated, the plasmon nonlocality for a single graphene sheet on a metal substrate in our model calculations yields dispersion curves which compare well qualitatively with reported experimental data, accounting for the plasma mode frequencies, their intensity and damping. We even pointed out that damping-free regions are modified with the presence of a finite gap which may be taken advantage of in the design and applications of plasmonic materials.

Finally, apart from all our reported results, there is another finding which, in our opinion, deserves highlighting and it is as follows. When the half-space conductor and 2D layer are not Coulomb-coupled, we normally obtain two plasmon branches, one associated with the 2D layer (acoustic plasmon, starting at the origin) and the other branch is associated with the surface plasmon, originating at 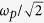. If the Coulomb interaction between the layer and the conductor is sufficiently strong and the energy bandgap is introduced, the upper “surface” plasmon acquires interesting features (being either damped or undapmed, depending on the energy gap; it could be divided into two undamped branches for certain gap values), as it was reported for graphene. So the plasmon branches are hybridized, and the surface plasmon demonstrates the behavior of a “layer-attributed” one.

The important conclusions of our work are as follows. We formulated and exploited a newly derived expression for plasmon dispersion in a superlattice of 2D layers which are Coulomb-coupled to a metallic substrate by taking into account the full nonlocality of the layers as well as the underlying conductor. We predict the existence of low-frequency nonlocal plasmon excitations at the vacuum-surface interface for various conditions of surface-layer separation as well as the energy gap. When the separation between the conducting surface and the nearest layer is less than some critical distance *d*_*c*_, the surface plasmon may not exist in the long wavelength limit. We obtain a surface plasmon at both intermediate as well as long wavelengths as this layer-surface separation is increased. For this hybrid structure, the surface plasmon frequency lies below the surface plasmon frequency for the semi-infinite substrate. Experimental verification of these simulated results may be achieved using high-resolution electron-energy-loss spectroscopy (EELS)^48^, for example. This paper was inspired by recent experimental work investigating the effect due to a metal on the collective plasmon mode of a single layer of graphene^9^,^10^. We presented a new approach for generating a tunable surface plasmon using hybrid semiconductors. Additionally, our proposed approach based on hybrid semiconductors can be generalized to include other novel two-dimensional materials, such as hexagonal boron nitride, molybdenum disulfide and tungsten diselenide.

## Additional Information

**How to cite this article**: Gumbs, G. *et al*. Plasmon Excitations of Multi-layer Graphene on a Conducting Substrate. *Sci. Rep*. **6**, 21063; doi: 10.1038/srep21063 (2016).
